# Spirochetal Lipoproteins and Immune Evasion

**DOI:** 10.3389/fimmu.2017.00364

**Published:** 2017-03-29

**Authors:** Alexei Christodoulides, Ani Boyadjian, Theodoros Kelesidis

**Affiliations:** ^1^David Geffen School of Medicine at University of California Los Angeles, Los Angeles, CA, USA

**Keywords:** spirochetes, lipoproteins, evasion mechanism, immune system, immunity

## Abstract

Spirochetes are a major threat to public health. However, the exact pathogenesis of spirochetal diseases remains unclear. Spirochetes express lipoproteins that often determine the cross talk between the host and spirochetes. Lipoproteins are pro-inflammatory, modulatory of immune responses, and enable the spirochetes to evade the immune system. In this article, we review the modulatory effects of spirochetal lipoproteins related to immune evasion. Understanding lipoprotein-induced immunomodulation will aid in elucidating innate pathogenesis processes and subsequent adaptive mechanisms potentially relevant to spirochetal disease vaccine development and treatment.

## Introduction

Spirochetes cause many human diseases such as syphilis, Lyme disease, and leptospirosis that pose major threats to public health (1). Epidemiological studies have shown that the incidence of Lyme disease (2–4), syphilis (5–7), and leptospirosis (8, 9) have increased, both within United States and globally (10, 11). However, the immunopathogenesis of spirochetal diseases remains unclear (12–14). Despite the apparent immune response generated following spirochete infection (i.e., tissue inflammation) (15), spirochetes are known to persist in their host (16) through a wide variety of mechanisms ranging from a dynamic outer membrane capable of antigenic variation in the presence of outer-surface proteins capable of inhibiting macrophage facilitated phagocytosis (17, 18).

A critical question is what cellular components can trigger the strong immune responses that are characteristic of spirochetal infections. Spirochetal membranes play a pivotal role in interacting with a host’s immune system (19, 20). Bacterial components such as lipopolysaccharides (LPSs) often play a major role in the induction of inflammation in bacterial infections (21, 22). Interestingly, aggressive immune responses are often observed despite the lack of LPS (endotoxin) in particular spirochetes, such as *Borrelia burgdorferi* (19, 23–25). Certain spirochetes such as *Treponema pallidum*, the spirochete responsible for syphilis, rely greatly on their ability to express adhesins over the surface of their membrane as a tool with which they can invade various tissues (26). Lipids compose 25–30% of a cell’s dry weight (19, 20). Detergent treatments of spirochetal membranes have confirmed that lipoproteins are the most abundant in number out of all proteins expressed by spirochetes (27–32) and are major integral spirochetal membrane proteins (27, 33). For example, *B. burgdorferi* species express >100 lipoproteins (34) and *Leptospira* spp. have >140 lipoprotein genes (35). Although numerous examples of spirochetal lipoproteins can be listed, a few prominent ones include OspA from *B. burgdorferi*, Tp47 from *T. pallidum*, and Lip32 from the *Leptospira* species (36–38). The number of bacterial lipoproteins that have been studied parallels the myriad of roles that lipoproteins play in bacteria such as envelope biogenesis, stress responses, pathogenicity, and nutrient transport (39–41).

However, there is limited evidence regarding the interplay between lipoproteins and human immune responses, partly due to the fact that *in vitro* studies do not accurately reflect human models. Understanding lipoprotein-induced immunomodulation will aid in elucidating innate pathogenesis processes and subsequent adaptive mechanisms potentially relevant to spirochetal disease vaccine development and treatment. In this article, we review the scientific evidence regarding the modulatory effects of spirochetal lipoproteins related to immune activation and evasion.

## Modulatory Effects of Spirochetal Lipoproteins Related to Activation of the Immune System

Understanding the dualistic roles (activation vs inhibition) of lipoproteins in their interaction with the immune system is pivotal (42). Thus, before we explore mechanisms of spirochetal immune evasion, a better understanding of all the regulatory mechanisms (such as pro-inflammatory effects and immune activation) of spirochetal lipoproteins is needed. Better understanding of spirochetal lipoproteins and their regulatory mechanisms may provide insight into clinical outcomes arising from spirochetal infections. For example, spirochetal infections may increase the risk of Alzheimer’s disease (43).

### Spirochetal Lipoproteins Induce Pro-inflammatory Effects

One of the primary manifestations of spirochetal infection is tissue inflammation that is the mainstay of spirochetal diseases such as Lyme neuroborreliosis (22, 29). Spirochetal lipoproteins are known to induce strong pro-inflammatory responses in their hosts (27, 33, 34, 44–52) that comprise the initial innate immune response to the invading pathogen (49). Components of the inflammatory infiltrate include keratinocytes, macrophages, leukocytes, and cells capable of responding to the presence of lipoproteins (53–55). A better understanding of the modulatory effects of spirochetal lipoproteins in myeloid and non-myeloid immune cells is needed.

### Spirochetal Lipoproteins Have Modulatory Effects on Neutrophils

Neutrophils have a major role in the immunopathogenesis of acute bacterial infections. Spirochetal lipoproteins, such as OspB, have been documented to inhibit neutrophil function and prevent oxidative burst in a variety of tissues, to prolong host infection (56–58). However, other lipoproteins can promote neutrophil activation. For example, OspA, even when presented at pico-molar concentrations, has been seen to play a role in the activation of neutrophils and their chemotaxic capabilities (51, 59). Subsequent to neurophil activation, neutrophil tissue infiltration contributes to localized tissue inflammation that is pre-dominant in inflamed arthritic joints and in myocarditis (associated with spirochetal infections) (50, 51, 60). In addition to mediating inflammatory responses, spirochetes, such as *Leptospira*, may induce neutrophils extracellular traps, which are a relatively novel pathogen-killing mechanism for extracellular microbes independent of phagocytic uptake and degranulation (61). Thus, spirochetal lipoproteins can modulate the function of neutrophils that are recruited early in acute inflammatory responses.

### Spirochetal Lipoproteins Have Pleotropic Modulatory Effects on Monocytes and Macrophages (M/M) That Are Mediated through Several Pathways

Except for neutrophils, M/M also play a major role in spirochetal immunopathogenesis. Lipoproteins bind CD14 in the membrane of M/M at the CD14 site that also interacts with LPS (62–64). This interaction activates the NF-κB pathway and induces pro-inflammatory responses (62, 63, 65). In addition, unlike the membrane-bound CD14, soluble CD14 also allows the activation of non-myeloid cells (66). Furthermore, the pro-inflammatory effects of spirochetal lipoproteins are often mediated by toll-like receptors (TLR) (67–69). TLR signaling leads to increased production of numerous cytokines that induce pro-inflammatory responses (25, 47). Interestingly, TLR-deficient mice had exacerbated inflammation and increased spirochetal burdens, both of which were attenuated by impairing T cell responses (70). As a bodily response to the vast amounts of pro-inflammatory cytokines produced upon spirochetal lipoprotein presence, monocytes have also been seen to produce IL-10 upon being presented with *B. burgdorferi* lipoproteins (71–75). IL-10, unlike cytokines such as IL-1 and IL-12, is known to reduce inflammation *via* TLR-pathway downregulation and can therefore assist in combatting the spirochetal infection as well as any possible chronic effects such as arthritis (76, 77). The above was confirmed in recent mice studies that utilized a TLR2 agonist, Pam3CSK4, to induce IL-10 production which attenuated inflammatory response to *Leptospira* (78). Thus, spirochetal lipoproteins exert their pro-inflammatory effects through several pathways including CD14, TLR, and NF-κB signaling and induce both pro-inflammatory (such as IL-1) and anti-inflammatory cytokines (IL-10) production in myeloid cells such as M/M.

### Spirochetal Lipoproteins Induce Activation of Dendritic Cells

Similar to the activation of neutrophils, M/M, spirochetes also maintain the ability to activate other myeloid cells such as dendritic cells, key components in linking both the innate and adaptive immune system. Spirochetes activate cell adhesion molecules such as intercellular adhesion molecule 1 (ICAM-1), which then facilitate T-cell interactions and subsequent dendritic cell migration to lymph nodes for the mounting of an immune response (79, 80). In early stages of inflammation, lipoproteins in *T. pallidum* upregulate ICAM-1 and activate dendritic cells to mount immune responses (25, 46, 49, 81–84). Immune activation can also be induced upon spirochetal death or phagocytosis of spirochetes, both processes of which lead to further introduction of lipoproteins to the surrounding environment (80). The modulatory effects of spirochetal lipoproteins on dendritic cells are particularly important since dendritic cells play a major role in vaccine responses (discussed below).

### Chronic Modulatory Effects of Spirochetal Lipoproteins and Effects on Adaptive Immunity May Drive Pathogenesis of Spirochetal Diseases

Spirochetal lipoproteins may also play a role in the transition from the acute immune responses to the more chronic effects that characterize spirochetal diseases such as arthritis, peripheral neuropathy, numerous neurologic manifestations, and the vascular endothelial damage thought to underlie a significant portion of the chronic symptoms in spirochetal diseases (85–89). Although the exact mechanism of transition may not be well understood, lipoproteins may activate B-cells and T-cells, both of which are known to play major roles in long-term adaptive immunity (46, 47, 49–52). Further understanding of the exact transition process has major potential in terms of possibly delaying, or inhibiting, many of the debilitating chronic effects characteristic of numerous spirochetal infections.

## Modulatory Effects of Spirochetal Lipoproteins Related to Facilitation of Immune Evasion

**Figure 1:**
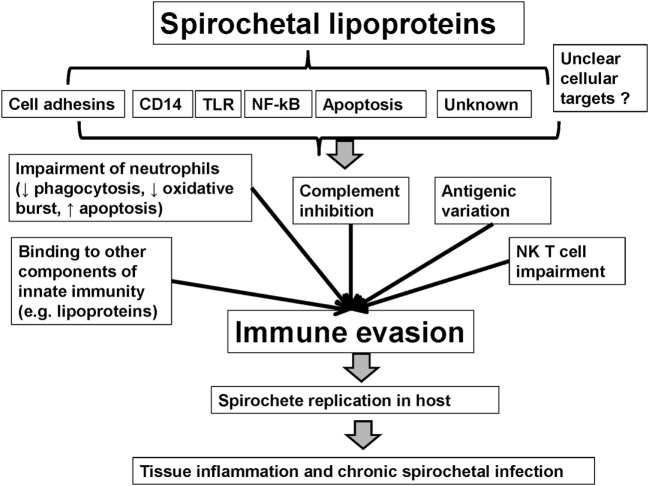
**Mechanisms of immune evasion mediated by spirochetal lipoproteins**.

Spirochetes evade a host’s immune system through mechanisms such as antigenic variation, which is capable of producing myriads of variants (90). Spirochetal interference of the innate immune system presents one more mechanism, in a list of many, to allow for the persistence of spirochetes in their host (16, 91). Spirochetes use multiple mechanisms of immune evasion that are related to spirochetal lipoproteins. Indeed, except for pro-inflammatory effects, lipoproteins are also responsible for modulatory effects such as immune evasion. Spirochetes may limit the expression of membrane lipoproteins and their access to antibodies (92, 93) or induce antigenic variation of surface lipoproteins (19, 90, 94–100). Spirochetal lipoproteins may also interact with, and inhibit, components of innate immunity such as the complement (63, 68, 88, 101–108), neutrophils, and serum lipoproteins (109). Major pathways of spirochetal immune evasion are discussed below (see also Table 1 and Figure 1) (110–130).

**Table 1:** **Mechanisms of immune evasion of major spirochetal lipoproteins**.

Bacteria	Role in immune evasion
*Borrelia burgdorferi*	Antigenic variation [VlsE proteins (118, 120, 131–134), OspC (135)]
	Evasion of complement-mediated lysis [OspE, Erp (136–138), CspA (139)]
	Impairment of neutrophil function (BBA57) (140)
Oral treponemes (*ex. Treponema denticola*)	C3b inactivation (various lipoproteins) (141)
*Borrelia recurrentis*	Antigenic variation (variable large and small protein genes and Vmp variants) (19, 110)
	Bind to complement regulatory proteins, i.e., CFH and CFHR-1 [FhbA, BhCRASP-1, and HcpA (142–145)]
*Borrelia turicatae*	Antigenic variation (variable large and small protein genes and Vmp variants) (19, 110)
	Inhibit C4bp and C1-Inh, the major inhibitors of the classical and lectin pathway of complement activation (CihC) (146)
	Binds to human complement regulators, Factor H, CFHR-1 (HcpA) (143)
*Borrelia hermsii*	Antigenic variation (variable large and small protein genes and Vmp variants) (19, 110)
	Bind to complement regulatory proteins, i.e., CFH and CFHR-1 [FhbA, BhCRASP-1, and HcpA (142–145)]
*Leptospira interrogans*	Impairment of neutrophil function (LIC11207) (147)
	Bind to complement regulators (LigA, LigB, Len A, Len B) (148)

*Antigenic variation in borrelias may result from recombination of variable large and small protein genes. Lipoproteins may also impair mechanisms of innate immunity such as neutrophil function and complement activation. These mechanisms allow the spirochete to evade the host’s immune response and persist in the mammalian host*.

*BBA57, *Borrelia burgdorferi* A57 protein; BhCRASP-1, *Borrelia hermsii* complement regulator-acquiring surface protein 1; C1-Inh, human C1 esterase inhibitor; CihC, C1-inhibitor and C4bp-binding protein; C4bp, C4b-binding protein; CspA, complement regulator-acquiring surface protein-1; Erp, OspE-F-related lipoprotein; FhbA, complement factor H-binding protein; HcpA, human complement regulator and plasminogen-binding protein; LIC11207, *L. interrogans* serovar Copenhageni (LIC) protein 11207; LigA, leptospiral immunoglobulin-like protein A; LigB, leptospiral immunoglobulin-like protein B; OspC, outer-surface protein C; OspE, outer-surface protein E; VlsE, variable major protein-like sequence E; Vmp, variable major lipoprotein*.

### Differential Dynamics of Spirochetal Lipoprotein Expression As a Mechanism of Immune Evasion

The expression of lipoproteins on the outer leaflet of the membrane allows the spirochete to interact with tissues and the host’s immune system (110). Naturally, the vast abundance of lipoproteins a given spirochete can express are not all necessary at a given time point, and their expression is time sensitive (111). Although more work is needed to elucidate the time-sensitive expression of surface lipoproteins, studies have hinted at the possibility of a temperature-sensitive mechanism to underlie expression patterns (112). For example, OspA in *B. burgdorferi* is not needed upon host infection and is therefore downregulated upon infection of a host *via* a temperature-sensitive alteration in membrane composition (111). Coupled closely with the need of a lipoprotein to be expressed on the exterior of the cell for interactions to occur, the lipoprotein must maintain its N-terminus as it has been documented that it is this region specifically to which immune system–spirochete interactions occur (113, 114). In line with the above statement, removal of the N-terminus disrupts the aforesaid interactions while synthesis of N-terminus analogs restored immune cell activation (114, 115). The limitation of outer-membrane lipoprotein expression in spirochetes may also act as a mechanism to facilitate host humoral defense evasion. Antibody recognizable lipoproteins may be scarcely expressed on the exterior leaflets, as opposed to the relatively more lipoprotein dense cytoplasmic leaflet (92, 93, 116). Further studies are needed to elucidate the role of differential dynamics of spirochetal lipoprotein expression in spirochetal immunopathogenesis.

### Antigenic Variation of Surface Lipoproteins

Coupled with the limited expression of outer-membrane lipoproteins in spirochetes, antigenic variation is a major mechanism by which invading bacteria can evade the host immune response (117). Spirochetes also undergo a process of antigenic variation in terms of expressed outer-leaflet lipoproteins (96, 118). Studies in immunocompromised hosts have suggested that the host immune responses have a major role in producing spirochetal antigenic variants (96). Antigenic variation in borrelias may result from recombination of variable large and small protein genes (98) and the diversity of variable major lipoprotein lipoproteins allows these pathogens to evade the host immune response (19, 23, 119). Moreover, outer-leaflet lipoprotein variation also allows spirochetal adherence to a wide variety of host cells, as studies of *T. pallidum* TP0435 isoforms have recently shown (26). The antigenic variation of major surface lipoproteins is described in Table 1 (19, 90, 94–100).

The ability to vary surface lipoprotein expression has been studied in *B. burgdorferi*, where it has been shown that prolonged infections are due to the embodiment of a *vls* locus that is capable of random segmental variation in the surface-exposed lipoprotein it encodes (118, 120). The *vls* locus variation specifically allows for the variation in the encoded variable major protein-like sequence lipoprotein which has been documented to allow for persistence of *B. burgdorferi* in its host (120). The antigenic variation of spirochetes leads to evasion of the immune system and ultimately to the phenomenon of host relapsing (121). Most interestingly, antigenic variation characteristic of *B. burgdorferi* is only seen during host infection. Spirochetal antigenic variation has not been described *in vitro*. Thus, the cross talk between host cellular responses and *B. burgdorferi* is needed for development of antigenic variation (perhaps through downregulation of OspA) (96). Elimination of the ability to undergo antigenic variation, as was done in *Borrelia hermsii*, may greatly reduce host infectivity/persistence (119). Understanding the exact mechanisms behind a spirochete’s ability to elicit immune evasion *via* antigenic variation could set the basis for targeted interventions to inhibit infections (122).

### Inhibition of Neutrophil Function by Spirochetes

Neutrophil-mediated phagocytosis of pathogens is a major host immune response to infection. Thus, spirochetes evade immune responses by inactivating neutrophil function (56). The most prominent examples of the above can be seen with the *B. burgdorferi* surface protein OspB, which may prevent phagocytosis of the spirochete and inhibit respiratory/oxidative burst in a variety of tissues, such as the skin (56–58). It should be noted that *B. burgdorferi* also contains outer-surface protein C which plays a role in inhibiting phagocytosis by macrophages (18). Similar to OspB that impairs neutrophil function, the novel lipoprotein *Leptospira interrogans* serovar Copenhageni (LIC) protein 11207 from *Leptospira*, promotes apoptotic pathways in neutrophils (123). Thus, spirochetal lipoproteins can both activate and impair neutrophils.

### Lipoprotein Inhibition of Complement Activation

One of the major components of a host’s innate immune system is the complement system that plays a role in the phagocytosis/elimination of a pathogen and is a target of spirochetes upon infection (124). Activation of the complement system is known to occur through the recognition of surface-exposed lipoproteins as well as other antigens such as oligosaccharides (124). The multi-stage process of complement activation presents spirochetes (such as *B. burgdorferi*) with the opportunity to attack at multiple phases. For example, *B. burgdorferi* binds and inhibits the C1 initiation complex and accelerates C3b inactivation (91, 125). Furthermore, *B. burgdorferi* can bind either Factor H or FHL-1, two important complement regulators which upon being bound by CRASP-2 and CRASP-1 (*B. burgdorferi* membrane-bound lipoproteins), respectively, are inactivated and inhibit formation of complement system activation products (126, 127). *B. burgdorferi* also maintains the ability to bind factor H, *via* particular Osp, such as outer-surface protein E, accomplishing the same outcome as with CRASP-2 binding (128). Hijacking of the complement system is a conserved mechanism of immune evasion among numerous pathogens (such as *Plasmodium falciparum*) (129). Therefore, understanding the mechanisms behind complement hijacking in spirochetes could potentially contribute to understanding conserved pathways in other pathogens.

### Lipoprotein Inhibition of Natural Killer T (NKT) Cells

Natural killer (NK) cells act to bridge the innate and adaptive immune responses to pathogenic infections; however, it is their ability to respond to a variety of lipid antigens that allows them to maintain a functional presence during combat of spirochetal infections (130). Spirochetes are capable of interfering with the NKT cells that respond to CD1d glycolipids on the surface of spirochetes such as *B. burgdorferi* (149). Although the exact biochemical pathway of interference is not well understood, patients with syphilis have been known to exhibit low NKT numbers (150). Further studies are needed to understand the possible interaction between spirochetal lipoproteins and NK cells.

## Understanding Lipoprotein-Mediated Pathways of Immune Evasion may Pave the Way for Development of Strategies to Treat Spirochetal Infections

Understanding the pleotropic modulatory effects of lipoproteins may contribute to the development of new approaches to combat a plethora of diseases (151–154). Use of adjuvants in vaccines may enhance recognition of whole proteins by the adaptive immune system (151, 155). The immunopotent effects of spirochetal lipoproteins have hinted at the possibility for the development of vaccines that rely on the use of synthetic or derived lipopeptides (151, 155, 156). Spirochetal lipoproteins, such as OspA, can be expressed on the surface of outer-membrane vesicles to elicit an immune response similar to vaccines (157). Improvements in recombinant bacterial lipoprotein generation promise to make lipopeptide-based vaccines more feasible in the near future (158). The incorporation of numerous epitopes, such as lipoproteins, as adjuvants into vaccines can help target various diseases including cancer (155, 159). On the other hand, incorporation of a lipid moiety in peptide-based vaccines may induce TLR2 signaling in dendritic cells and subsequent protection against viral and bacterial infections (156). Finally, the use of lipopeptide-based antibiotics such as daptomycin, that can cause both immunomodulation (160) and also target spirochetes (161), remains to be studied as a therapeutic option for patients with spirochetal infections.

## Conclusion

Lipoproteins play a significant role in the various stages of a spirochete’s ability to infect a host and survive, through pleotropic effects involving transfer from vector to host, immune activation, or even immune evasion. Further studies are needed to understand the molecular basis and mechanisms that underpin the numerous modulatory effects (both acute and chronic) of spirochetal lipoproteins. The payout from such targeted research can be significant considering the sheer amount of spirochetal infections occurring on a yearly basis as well as the morbidity associated with chronic spirochetal infections in humans. Ultimately, the use of knowledge surrounding spirochetal lipoproteins can be put toward the development of vaccines or, perhaps shed light on the pathogenesis of other vector-based pathogens.

## Author Contributions

AC, AB, and TK contributed to writing of this manuscript.

## Conflict of Interest Statement

The authors declare that the research was conducted in the absence of any commercial or financial relationships that could be construed as a potential conflict of interest.
